# Synonymous Genetic Variation in Natural Isolates of *Escherichia coli* Does Not Predict Where Synonymous Substitutions Occur in a Long-Term Experiment

**DOI:** 10.1093/molbev/msv161

**Published:** 2015-07-20

**Authors:** Rohan Maddamsetti, Philip J. Hatcher, Stéphane Cruveiller, Claudine Médigue, Jeffrey E. Barrick, Richard E. Lenski

**Affiliations:** ^1^Ecology, Evolutionary Biology, and Behavior Program, Michigan State University; ^2^BEACON Center for the Study of Evolution in Action, Michigan State University; ^3^Department of Computer Science, University of New Hampshire; ^4^CNRS-UMR 8030 and Commissariat à l'Energie Atomique CEA/DSV/IG/Genoscope LABGeM, Evry, France; ^5^Department of Molecular Biosciences, Institute for Cellular and Molecular Biology, Center for Systems and Synthetic Biology, The University of Texas at Austin

**Keywords:** experimental evolution, genetic variation, mutation rate, effective population size

## Abstract

Synonymous genetic differences vary by more than 20-fold among genes in natural isolates of *Escherichia coli*. One hypothesis to explain this heterogeneity is that genes with high levels of synonymous variation mutate at higher rates than genes with low synonymous variation. If so, then one would expect to observe similar mutational patterns in evolution experiments. In fact, however, the pattern of synonymous substitutions in a long-term evolution experiment with *E. coli* does not support this hypothesis. In particular, the extent of synonymous variation across genes in that experiment does not reflect the variation observed in natural isolates of *E. coli*. Instead, gene length alone predicts with high accuracy the prevalence of synonymous changes in the experimental populations. We hypothesize that patterns of synonymous variation in natural *E. coli* populations are instead caused by differences across genomic regions in their effective population size that, in turn, reflect different histories of recombination, horizontal gene transfer, selection, and population structure.

## Introduction

According to the neutral theory of molecular evolution, mutation and random genetic drift are largely responsible for shaping the patterns of genetic variation in nature (Kimura 1968). The generality of the empirical predictions of this theory remains contentious (Hahn 2008), but it does provide a useful quantitative framework for generating falsifiable hypotheses. One of the central predictions of neutral theory is that synonymous variation in protein-coding sequences should reflect the underlying mutation rate and the time passed as populations diverge.

**Fig. 1.**
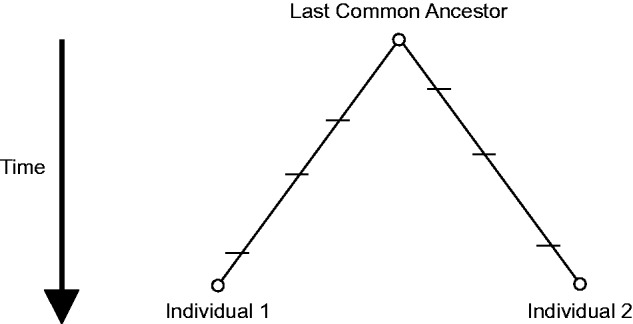
The expected time to coalescence for individuals from an evolving haploid population is *N*_e_ generations. Tick marks show neutral mutation events along two lineages, which occur at some rate µ per generation. The expected number of mutations separating Individuals 1 and 2 is 2*N*_e_µ. If all genes in the genome have experienced the same *N*_e_, then significant variation among genes in the per-site rate of accumulation of neutral mutations would imply gene-specific heterogeneity in the underlying mutation rate.

Based on patterns of synonymous variation across the genomes of diverse *Escherichia coli* isolates, Martincorena et al. (2012) hypothesized that natural selection has optimized local mutation rates such that physiologically important, highly expressed genes that experience strong purifying selection mutate at lower rates than less important, lowly expressed genes. They used synonymous nucleotide diversity, θ_s_, to estimate the mutation rate for each gene. The expected number of neutral mutations in a given lineage is *t*µ, where *t* is time in generations and µ is the mutation rate over the relevant genomic sites. The expected divergence time (i.e., the time to coalescence) between two lineages is *N*_e_ generations. Therefore, the expected number of neutral mutations separating two sampled genomes is 2*N*_e_µ (fig. 1). In their analysis, Martincorena et al. (2012) implicitly assumed that all of the genes in the core genome of *E. coli* have experienced the same coalescence time and effective population size, so that any significant variation among those genes in the quantity θ_s_ was attributed to differences in gene-specific mutation rates. They excluded noncore genes from their analysis owing to the recognition that such genes probably have different coalescence times as the result of horizontal transfer between species.

The hypothesis of local optimization of mutation rates comes from Martincorena et al. (2012), but their empirical findings of nonrandom patterns in synonymous substitutions find precedence in earlier studies. Comparing *E. coli* and *Salmonella typhimurium* gene sequences and controlling for gene expression, Sharp et al. (1989) found a significant relationship between synonymous divergence and distance from *oriC*, the chromosomal origin of replication. They proposed that genes farther from *oriC* tend to experience higher mutation rates than those closer to *oriC* because genes closer to *oriC* have higher copy numbers in growing cells and therefore more opportunity for recombination-based repair. Eyre-Walker (1994) reported that synonymous substitutions tend to be clustered in genomes, and he proposed several possible explanations: Template-based mutational events that can introduce multiple base-pair changes, interspecific recombination, and selection acting on the secondary structure of nucleic acids.

As in many bacterial species, gene content varies substantially among *E. coli* strains. In a sample of 20 *E. coli* genomes, approximately 18,000 different genes were found in at least one strain, whereas only approximately 2,000 were found in all 20 strains (Touchon et al. 2009). The latter set of genes forms the core genome of *E. coli*, and the synonymous variation in that core genome is the subject of the study by Martincorena et al. (2012) as well as our own.

If the point-mutation rate varies across the *E. coli* genome, and in particular if mutations are selectively neutral, then we should see neutral mutations accumulate at different rates across different genes in evolution experiments. On the timescale of experiments with large asexual populations that begin without any standing variation, increases in the frequency of synonymous mutations should occur almost entirely when they hitchhike with beneficial driver mutations. Because beneficial mutations will also drive neutral mutations in other backgrounds extinct, the net effect is a wash—that is, comparing two asexual genomes separated by *t* generations, there will have been *t* opportunities for any given neutral mutation to occur, regardless of whether other mutations were under selection. Therefore, the expected rate of accumulation of neutral mutations should reflect their underlying mutation rate. Not all synonymous changes are perfectly neutral, but any fitness effects they have—even if beneficial—are generally far too small for these mutations to increase in frequency on their own over the course of even the longest experiment (Wielgoss et al. 2011). In fact, even strongly beneficial mutations rarely fix alone during experiments with large asexual populations owing to a phenomenon called clonal interference (Lang, Rice, et al. 2013; Maddamsetti et al. 2015). Clonal interference occurs because, in the absence of recombination, beneficial mutations that arise in different lineages compete with one another, thus slowing the progress of each one toward fixation (Gerrish and Lenski 1998; Barrick and Lenski 2013). As a consequence, only the most highly beneficial mutations can drive selective sweeps in the clonal interference regime (Levy et al. 2015), and secondary beneficial mutations that arise after the contending lineages reach high frequencies often determine which lineage ultimately prevails (Woods et al. 2011; Maddamsetti et al. 2015). Most synonymous mutations, even if they are not perfectly neutral, should have very small selection coefficients; as a consequence, they will have a negligible effect on the fixation probability of lineages that also have beneficial mutations with much larger fitness effects. Therefore, the rate of accumulation of synonymous substitutions—whether they are strictly neutral or not—provides a reasonable proxy for the point-mutation rate in evolution experiments.

From mutation-accumulation experiments and fluctuation tests, it is clear that both the rate and spectrum of spontaneous mutations vary across the tree of life (Luria and Delbrück 1943; Keightley et al. 2009; Ossowski et al. 2010; Lee et al. 2012; Sung et al. 2012; Ford et al. 2013). Multiple studies have reported variation in the mutation rate depending on chromosomal location, local sequence context, and chromatin structure (Sharp et al. 1989; Lang and Murray 2008, 2011; Warnecke et al. 2012; Foster et al. 2013; Long et al. 2015). Also, the process of transcription has been reported to be mutagenic (Kim and Jinks-Robertson 2009; Park et al. 2012; Paul et al. 2013).

Other patterns where substitution rates vary with chromosomal location have been seen elsewhere. In *Burkholderia* and *Vibrio* species that have primary and secondary chromosomes, genes on the secondary chromosome have higher rates of both nonsynonymous and synonymous substitutions (Cooper et al. 2010). This variation appears to indicate that fast-evolving genes have disproportionately migrated to the secondary chromosome. This finding also raises the possibility that selection has operated on the rate and spectrum of mutations in such a way that more important genes mutate less often.

Natural selection can also shape synonymous variation directly. For example, purifying selection on synonymous sites has been seen in *Drosophila melanogaster* (Lawrie et al. 2013), and synonymous substitutions that are beneficial because they increase gene expression have been reported in an evolution experiment with *Pseudomonas fluorescens* (Bailey et al. 2014). Natural selection also affects codon usage, and recoding a functionally important gene through synonymous changes can affect fitness (Agashe et al. 2013). Other more indirect evidence also implicates selection as an important force shaping synonymous variation in bacteria. Although mutation is universally biased toward increased AT-content in bacteria, genomic GC-content varies among species from less than 20% to more than 70%. GC-content at synonymous sites strongly correlates with genomic GC-content; the fact that genome composition is inconsistent with the mutational bias suggests that selection has acted in opposition to the mutational bias even at synonymous sites (Hershberg and Petrov 2010; Hildebrand et al. 2010; Rocha and Feil 2010).

In this article, we confirm the finding by Martincorena et al. (2012) that synonymous nucleotide diversity varies by more than an order of magnitude across the core genome of *E. coli*. In other words, some gene trees have much longer branches, on average, than other gene trees, even in the core genome. This result means that different genes give different estimates of when *E. coli* isolates diverged from each other, assuming that mutation rates do not vary across the genome. However, Martincorena et al. argued instead that this heterogeneity is caused by local genomic differences in the mutation rate. If their hypothesis were correct, then we would expect such mutation-rate heterogeneity to occur and be evident in the long-term evolution experiment (LTEE) with *E. coli* that has been running for more than 25 years (Lenski and Travisano 1994; Wiser et al. 2013). To test that prediction, one must focus on the effects of mutation rate rather than natural selection. To that end, we count the number of synonymous substitutions that have accumulated in almost 3,000 genes after 40,000 generations in clones (i.e., individuals) from 12 replicate populations, while also controlling for gene length. Most of the synonymous mutations occurred in populations that evolved hypermutator phenotypes owing to defects in DNA repair (Sniegowski et al. 1997; Wielgoss et al. 2013). However, we will show that the base substitution signatures of different types of hypermutability do not affect our results.

In brief, we find no evidence from these experimental populations that those core genes with low synonymous nucleotide diversity in nature have lower mutation rates than those with high synonymous nucleotide diversity. Instead, we find a close correspondence between the number of synonymous substitutions in different genes and the length of those genes, consistent with the null hypothesis of a point-mutation rate that is homogeneous across the genome. We also find a weak, positive relationship between a gene’s level of expression and its rate of synonymous substitution, but this relationship is not significant when controlling for gene length; that is, longer genes tend to have slightly higher gene expression levels but also more sites at risk for mutation.

## Results

**Fig. 2.**
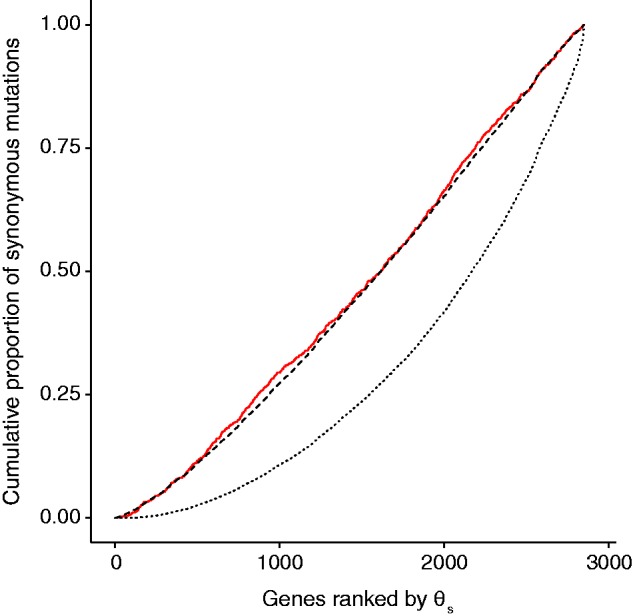
Synonymous substitutions observed in experimental populations of *Escherichia coli* closely match the null hypothesis of a uniform point-mutation rate across genes, but not the distribution expected if the variability in θ_s_ across genes in natural isolates is explained by gene-specific differences in the point-mutation rate. Each observed or hypothetical series shows the cumulative proportion of 1,069 synonymous substitutions in 2,834 genes that have been sorted and ranked by their θ_s_ values (i.e., the synonymous nucleotide diversity seen in natural isolates for each gene). The red line shows the observed distribution of synonymous mutations in 12 independently evolved genomes after 40,000 generations. The dashed curve shows the null hypothesis of a uniform point-mutation rate, where gene length alone predicts the occurrence of synonymous changes. The dotted curve shows the alternative hypothesis where each gene’s point-mutation rate is proportional to θ_s_.

We identified a total of 1,069 synonymous substitutions in the core genome (described in the Materials and Methods) of clones sampled from 12 independently evolved populations after 40,000 generations of the LTEE (Lenski and Travisano 1994; Wiser et al. 2013). To control for variation in gene length, we compared the observed cumulative distribution of synonymous substitutions across this gene set with the distribution expected under the null hypothesis of a uniform point-mutation rate (fig. 2). Despite a large number of events, there is no significant difference between the observed distribution and the null hypothesis (Kolmogorov–Smirnov test, *D* = 0.0281, *P* = 0.21). In broad terms, therefore, the accumulation of synonymous mutations in the LTEE is consistent with a uniform rate of point mutation across the *E. coli* genome.

**Fig. 3.**
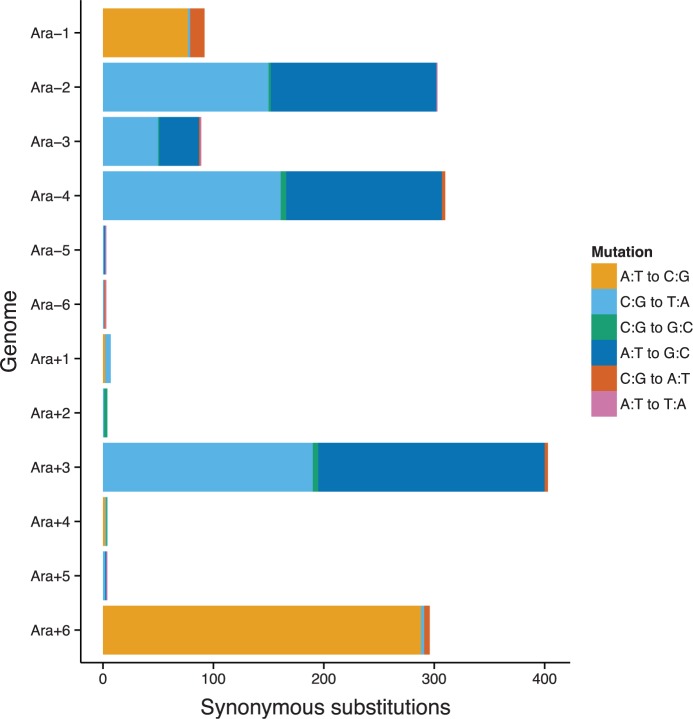
Hypermutator clones have distinctive spectra of synonymous mutations in addition to elevated mutation rates. Clones with defective *mutS* or *mutL* genes (Ara−2, Ara−3, Ara−4, Ara+3) have large numbers of C:G to T:A and A:T to G:C transitions, whereas clones with defects in *mutT* (Ara−1, Ara+6) have large numbers of A:T to C:G transversions.

Under the alternative hypothesis, the variation among genes in the quantity θ_s_ reflects differences in their underlying mutation rates. In that case, we would expect µ—and thus the distribution of synonymous mutations in the evolution experiment—to be directly proportional to θ_s_. However, the difference between that expectation and the distribution of synonymous mutations observed in the evolution experiment is extremely significant (fig. 2; Kolmogorov–Smirnov test, *D* = 0.244, *P* < 10^−^^15^). Importantly, this difference holds when clones from the four mismatch-repair (*mutS* or *mutL*) and two base-excision repair (*mutT*) hypermutator lineages are analyzed separately (*P* < 10^−^^15^ and *P* < 10^−^^8^, respectively). Hence, rejection of this hypothesis does not depend on the particular mutational signature of one or the other class of hypermutator (fig. 3). None of these results changes when we use the θ_s_ estimates from Martincorena et al. (2012) instead of our own estimates. Therefore, the data from the LTEE do not support the hypothesis of Martincorena et al. (2012) that the mutation rate has been locally optimized. Instead, the point-mutation rate is remarkably uniform across the core genome (fig. 2). Of course, we cannot prove that such uniformity would persist if we had equally large samples of synonymous changes from nonmutator populations. It is possible that the hail of mutations caused by hypermutability obscures subtle differences among genes in their point-mutation rate; for example, defects in mismatch repair in yeast mask variation in the mutation rate associated with replication timing (Lang and Murray 2011; Lang, Parsons, et al. 2013). Nevertheless, the concordance of the results across two functionally distinct classes of hypermutators indicates that the uniformity we observe in the location of synonymous changes is not a peculiar feature caused by one or the other affected mutational process. Furthermore, hypermutators occur in natural *E. coli* populations, and there is evidence of recurrent losses and reacquisitions of functional DNA repair genes (including *mutS*) during *E. coli* evolution (Denamur et al. 2000). Thus, hypermutators likely also contribute to the natural sequence variation analyzed by Martincorena et al. (2012), although the extent of this contribution is unclear.

**Fig. 4.**
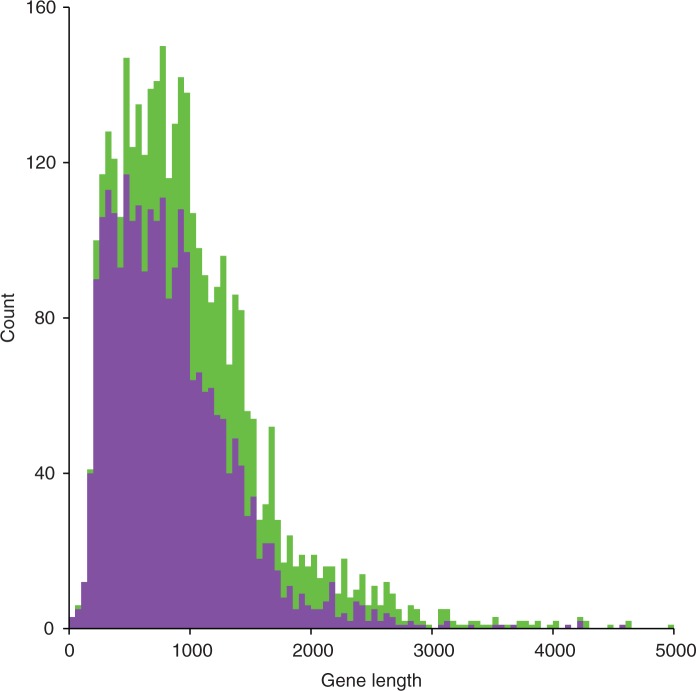
Synonymous substitutions tend to be found in longer genes. Genes with at least one synonymous substitution after 40,000 generations (green) are on average 1,296 bp long, whereas those without any synonymous substitutions (purple) are on average only 850 bp long. The bin width is 50 bp.

After seeing an earlier version of our analysis above, Martincorena and Luscombe (2012) pointed out that the presence of synonymous substitutions in the LTEE seems to be correlated with gene expression. To examine this issue and its relevance to the issue at hand, we grouped all of the genes from the LTEE ancestral genome into two categories: Those with a synonymous substitution in at least one of the 12 evolved genomes, and those without any synonymous substitutions. As indicated in the Materials and Methods, we used gene expression data obtained under the same conditions as used in the LTEE (Cooper et al. 2003), because those expression levels would be the ones relevant to any effect on mutation rate in our study. Indeed, there is a small (3.7%) but significant difference in mean gene expression between these two sets of genes (Welch’s *t*-test, *P* = 0.0137). However, gene expression itself is also weakly correlated with gene length (*r* = 0.09), so the difference in expression between genes with and without synonymous changes might be driven by gene length. To examine that possibility, we calculated Kendall’s partial coefficient of rank-correlation between synonymous substitutions and gene expression controlling for gene length (Kendall 1942), and we tested its significance assuming normality (Kim and Yi 2006). In fact, the relationship between gene expression and synonymous mutations is not significant (*P* = 0.73) when gene length is taken into account. On average, genes with synonymous substitutions are 1,296 bp long, whereas genes without synonymous substitutions are only 850 bp long (fig. 4), and this difference is highly significant (Wilcoxon rank-sum test, *P* < 10^−^^15^). Taken together, these analyses clearly show that gene length is the main factor determining where synonymous substitutions have accumulated in the genomes of the LTEE populations.

## Discussion

Martincorena et al. (2012) proposed that natural selection has optimized point-mutation rates at the level of genes within genomes, such that more important genes mutate at lower rates than do less important genes. They used the gene-specific level of synonymous nucleotide variation in diverse *E. coli* isolates to estimate the mutation rate for each gene. On theoretical grounds, Chen and Zhang (2013) argued that the locally optimized mutation rate hypothesis is untenable, owing to the extremely small size of the relevant selection coefficients, and they presented empirical evidence that also cast doubt on the hypothesis. Moreover, we now show that the accumulation of synonymous changes—a proxy for the underlying mutation rate—in a LTEE with *E. coli* is extremely well correlated with gene length, but not with the extent of synonymous diversity in natural isolates (fig. 2). Taken together, these theoretical and empirical considerations indicate the need for some alternative explanation to explain why synonymous diversity varies so much across the core genome of *E. coli*.

In general, synonymous nucleotide diversity in natural populations depends not only on the mutation rate but also on the effective population size, which in turn depends on the rate and history of recombination and horizontal gene transfer (HGT). Intragenomic variation in effective population size has been found in many eukaryotic species (Gossmann et al. 2011). Furthermore, population structure can cause variation in effective population size (Nordborg 1997), including different histories of HGT at different loci. Research with *D. melanogaster* has shown that nucleotide diversity in natural isolates positively correlates with local recombination rates (Begun and Aquadro 1992), and this relationship has been seen in many other species including human, mouse, *Caenorhabditis elegans*, mosquito, *Arabidopsis thaliana*, and tomato (Hahn 2008). In a related vein, a population genomic analysis of *D. simulans* compared with sister species *D. yakuba* and *D. melanogaster* found that nucleotide diversity and divergence fluctuate on large scales across the genome; these fluctuations are probably related to natural selection, and not the mutation rate (Begun et al. 2007). If these fluctuations were caused by recombination being mutagenic, then nucleotide divergence between species should be positively correlated with recombination, which is not the case in the *D. simulans* data set. Instead, genomic regions with more recombination may allow polymorphic loci to escape the effects of selection at other sites (Hahn 2008). A recent study mapped recombination rates at fine scales over a significant portion of the *D. pseudoobscura* and *D. miranda* genomes, supporting the hypothesis that such patterns of nucleotide diversity are caused by recombination preserving variation that would otherwise be eliminated by selection operating at linked sites (McGaugh et al. 2012).

Empirical work has also shown the importance of recombination and HGT for microbial genome evolution, even over short timescales. In natural populations of *E. coli*, recombination between related strains generates substantially more nucleotide substitutions than does mutation (Guttman and Dykhuizen 1994; Dixit et al. 2015). A population genomic study of *Vibrio cyclitrophicus* found that recombination plays a fundamental role in ecological differentiation as positively selected genes, rather than entire genomes, sweep through evolving populations (Shapiro et al. 2012). Direct measurements of substitution rates in nature reveal the success of hybrid genotypes containing alleles from distinct *Leptospirillum* groups over mere decades (Denef and Banfield 2012). In Archaea, species are determined largely by ecological differentiation, rather than by physical or genetic barriers to gene flow (Cadillo-Quiroz et al. 2012). These empirical studies demonstrate that recombination and HGT play important roles in microbial evolution over short timescales. Moreover, simulations of evolving populations show that the topology of a bacterial phylogeny can be recovered in the presence of recombination, but the branch lengths can be badly distorted (Hedge and Wilson 2014).

In the study by Martincorena et al. (2012) and in our work, each *E. coli* genome sampled from nature contains information not only about the mutation rate that its ancestors experienced but also its particular history of recombination, HGT, and natural selection. This information is more or less distinct, depending on its genealogical history, from that contained in the other *E. coli* genomes, even if we consider only their shared core. Owing to ecological and genetic differences between strains and related species, some *E. coli* genes may be more readily transferred between diverged lineages than other genes, even among those genes that constitute the core genome. Indeed, experiments have shown that some genes—including those that encode ribosomal proteins often used as phylogenetic markers—are more resistant to HGT between species than others (Sorek et al. 2007). Also, computational work has shown that highly expressed genes tend to be more resistant to HGT (Park and Zhang 2012).

Recombination and HGT can also affect the evolution of mutation rates in interesting and important ways. First, recombination can directly impact mutation rates. Functional mismatch repair genes in natural isolates of *E. coli* show high sequence mosaicism relative to housekeeping genes, indicating that repair genes have undergone frequent HGT (Denamur et al. 2000). Second, recombination affects how selection operates on mutation rates, with even rare recombination reducing selection for hypermutable phenotypes (Tenaillon et al. 2000). Hypermutators often evolve in experiments with bacteria, presumably because they reduce the waiting time for new beneficial mutations, although at the cost of an increased load of harmful mutations (Sniegowski et al. 1997; Wielgoss et al. 2013); unlike in nature, however, the bacteria in these experiments lack the potential for HGT that could restore a functional gene from another strain.

These issues are important because they support the possibility that variation in θ_s_ among the core genes of *E. coli* reflects differences in their histories of recombination and HGT, rather than gene-specific differences in their mutation rates. Martincorena et al. (2012) showed that genes with low θ_s_ tend to have functional characteristics typical of housekeeping genes subject to strong purifying selection, and they used that as evidence to argue that mutation rates have been locally optimized. Their observations are also consistent with recombination and HGT, however, because highly conserved genes should also resist the influx of foreign alleles more effectively than genes that face weak or variable selection. In summary, our analyses offer no support for the hypothesis that point-mutation rates vary among genes and have been optimized, as postulated by Martincorena et al. Instead, we think a more plausible explanation is that the variation among genes in their synonymous diversity reflects different histories of recombination and HGT.

## Materials and Methods

### Calculating Synonymous Diversity for the Core Genome of *E. coli*

Using procedures described elsewhere (Cooper et al. 2010), we identified a total of 2,837 single-copy orthologous genes that were shared by all of the *E. coli* strains listed in table 1. We realize that three of the strains (REL606, BL21-DE3, and K-12-MG1655) have been in laboratories for many years, but the vast majority of their mutations accumulated in nature. We also recognize that two of them (REL606 and BL21-DE3) derive from the same natural isolate (Daegelen et al. 2009; Jeong et al. 2009), but that redundancy does not affect our substantive conclusions because we obtained essentially the same results when we replicated our analyses using the θ_s_ estimates from Martincorena et al. (2012). We used the SATé package (Liu et al. 2009) to align the gene sequences. We then performed the θ_s_ estimation procedure of Martincorena et al. (2012) for these alignments using OmegaMap (Wilson and McVean 2006). Using OmegaMap, we could estimate θ_s_ for 2,835 of the 2,837 single-copy orthologous genes; another gene did not pass a filter for pseudogenes and proteins containing selenocysteine. We consider the resulting set of 2,834 protein-coding genes to be the core genome for our study.

**Table 1.** *Escherichia coli* Genomes Used in This Study.

Strain	NCBI Accession	JGI Taxon ID	Size (bp)	Coding Sequences
*Escherichia coli* B str. REL606	NC_012967	644736359	4,629,812	4,404
*Escherichia coli* BL21(DE3)	NC_012892	646862324	4,558,947	4,360
*Escherichia coli* K-12, MG1655	NC_000913	646311926	4,641,652	4,140
*Escherichia coli* O157:H7 Sakai (EHEC)	NC_002695	637000108	5,498,450	5,204
*Escherichia coli* O26:H11 str. 11 368	NC_013361	648028025	5,697,240	5,528
*Escherichia coli* UMN026	NC_011751	644736365	5,202,090	4,819
*Escherichia coli* SMS-3-5	NC_010498	641522624	5,068,389	4,773
*Escherichia coli* HS	CP000802	640753025	4,643,538	5,228
*Escherichia coli* 536	NC_008253	637000104	4,938,920	4,553
*Escherichia coli* O111:H- str. 11 128	NC_013364	646311924	5,371,077	5,167

### Synonymous Substitutions in the LTEE

We identified all synonymous substitutions in the genome sequences of single clones isolated from each of the 12 independently evolved populations after 40,000 generations of the LTEE (Lenski and Travisano 1994; Wiser et al. 2013). Six of these clones derived from lineages that had evolved mutations in *mutS*, *mutL*, or *mutT* (Sniegowski et al. 1997; Barrick et al. 2009; Wielgoss et al. 2013). The genomic reads for all 12 populations have been deposited at the NCBI Sequence Read Archive where the accession numbers are SRP001369 (Barrick et al. 2009), SRP004752 (Blount et al. 2012), SRP045228 (Raeside et al. 2014), SRP060289 (Wielgoss et al. 2011), and SRP060314 (this study). Across the entire genome, we identified a total of 1,518 synonymous substitutions, which are summarized by population in figure 3. However, in our other analyses we used only the 1,069 synonymous substitutions present in the core genome, including 1,055 in the hypermutator lineages.

### Gene Expression Analyses

We compared the levels of gene expression in the ancestor between those genes that either had or lacked synonymous substitutions in any of the 12 experimentally evolved genomes. We used previously reported gene expression data that were measured under the same conditions as used in the LTEE (Cooper et al. 2003).

### Statistical Analyses, Computer Code, and Figures

The data and analysis scripts have been deposited in the Dryad Digital Repository (doi:10.5061/dryad.266g4).
